# Nutrition educational interventions for athletes related to low energy availability: A systematic review

**DOI:** 10.1371/journal.pone.0314506

**Published:** 2025-02-14

**Authors:** Alexandra F. DeJong Lempke, Laura M. Reece, Kristin E. Whitney

**Affiliations:** 1 Department of Physical Medicine and Rehabilitation, Virginia Commonwealth University School of Medicine, Richmond, Virginia, United States of America; 2 Institute of Women’s Health, Virginia Commonwealth University, Richmond, Virgnia, United States of America; 3 Division of Sports Medicine, Department of Orthopedics, Boston Children’s Hospital, Boston, Massachusetts, United States of America; 4 Harvard Medical School, Harvard, Massachusetts, United States of America; 5 Female Athlete Program, Boston Children’s Hospital, Boston, Massachusetts, United States of America; United States Olympic & Paralympic Committee, UNITED STATES OF AMERICA

## Abstract

Low energy availability (LEA) is a prevalent concern among athletes, often attributed to intentional or unintentional under-fueling behaviors. Nutritional and energy availability educational interventions are poised for successful LEA prevention, with a robust body of literature examining intervention effectiveness. Thus, this systematic review aimed to synthesize the available evidence on nutritional education interventions to address gaps in nutritional knowledge and combat LEA among athletes. Medical databases (MEDLINE, Web of Science) were systematically searched on July 11, 2023, and an updated search was conducted on July 26, 2024. Studies conducted among adult athletes who underwent nutritional education interventions with assessed effects on dietary knowledge, behaviors, and/or LEA outcomes were included. Study quality was assessed using the Physiotherapy Evidence Database (PEDro) scale by two blinded assessors. Intervention methodology and primary outcomes related to nutritional interventions were extracted by a single assessor. Twelve articles were included (mean PEDro score: 5). Interventions ranged from 1 to 20 sessions, and 10- to 120-minute durations. Most studies employed in-person educational sessions on fueling and macro- and micro-nutrient intake for athletic performance. Half of included studies included LEA education. Intervention approaches were largely heterogeneous, although most programs had favorable outcomes for nutrition knowledge and fueling behaviors. Nutrition interventions appear to be beneficial for athletes in the context of LEA, though current approaches are largely heterogenous. Future research should seek to develop a translational nutritional education plan for broad application in athletes designed to increase nutritional knowledge and combat LEA.

## Introduction

Low Energy Availability (LEA) refers to a prolonged and/or severe mismatch between energy intake and energy expenditure from physical activity, and results in insufficient energy stores to support basic physiological functioning and physical performance [1–5]. LEA is a prevalent concern affecting at least 1 in 4 athletes throughout sport participation, with higher rates noted among female athletes [6–12]. LEA can present on a spectrum, ranging from adaptable LEA, which refers to short-term LEA with mild and reversible effects, to problematic LEA, which may eventually be associated with physical and mental health declines and athletic performance deficits known as Relative Energy Deficiency in Sport (REDs) [4, 13, 14]. LEA and subsequent REDs affects multiple body systems, including but not limited to interruptions to the gastrointestinal, cardiovascular, reproductive, and musculoskeletal systems [1, 4]. The wide-ranging health concerns associated with LEA substantiate the need for viable, evidence-based clinical interventions to interrupt energy imbalances among athletes.

There are multiple potential catalysts to the development of LEA, such as disordered eating behaviors, diagnosed eating disorders, and intentionally or unintentionally under-fueling to support bouts of athletic training and competition [4]. Previous research has demonstrated that knowledge of LEA alone does not relate to LEA prevalence among athletic populations who already have the LEA condition, though it remains unclear if knowledge prior to condition development may be beneficial in combating LEA risk [15]. For example, it is possible that athletes may have obtained knowledge related to LEA following their own exploration into physical symptoms, or after seeking medical care. Thus, examining the utility of nutritional education prior to condition development are warranted. Past findings suggest that other factors may underpin behaviors contributing to future LEA development, such as the common misconception that excessive leanness contributes to optimal athletic performance, especially for endurance (i.e., distance running, swimming, cycling) and aesthetic sports (i.e., gymnastics, performing arts, wrestling) [16, 17]. These concepts are complex as, in some context, leanness within an individualized range has been associated with improved athletic performance, dependent upon the athlete and sport. However, this is often taken out of context and over-generalized as a “one-size-fits-all” model, when instead this dynamic is a complex balance and management within the individualized athlete profile. Thus, misconceptions and overreliance on inappropriate informational sources may ultimately contribute to psychological indicators of LEA, such as deliberate caloric restraint and an intrinsic drive for thinness [18, 19].

Another primary underlying factor to behaviors that result in LEA may be attributed to poor knowledge of ramifications of energy imbalance and under-fueling. For example, a previous study among marathoners identified that many individuals did not consult any resources for nutritional information (49.5%) or did not rely on official or reliable sources of nutritional information (i.e., only obtained recommendations from social media; 11.5%) [20]. Furthermore, respondents largely reported that they did not have an endurance training fueling strategy (14.3%), and did not plan on ingesting carbohydrates through food or sports drinks during a competitive race (24.0%) [20]. Similar findings have been identified among trail runners as about 50% of runners reported ingesting recommended carbohydrate fueling guidelines for endurance events [21]. While factors such as specialized diets (e.g., ketogenic diets) and experience may have factored into some of these past study findings, the prevalence of LEA across assessed athletes remained high (≥40% athletes) and cumulatively signals a broader problem in the athletic community [20–22].

Poor nutritional knowledge and/or planning for sport have also been identified among Australian rules football athletes with only about 55% of athletes answering questions correctly on sports nutrition specific questions [6], and among young female athletes as <50% of respondents answered correctly to questionnaires regarding LEA conditions and consequences [23, 24]. Athletes have also been found to depend on coaches for nutritional and/or LEA information, yet unfortunately objective knowledge in this domain among coaches remains low [24]. Ultimately, the lack of scientific nutritional information used among athletes or sole reliance on inappropriate educational sources or untrained professionals and concepts may perpetuate behaviors that render athletes susceptible to LEA. The onus is on clinicians, athlete health and performance teams, and other key stakeholders to provide adequate and evidence-based education and training on athletic nutrition. Previous research overwhelmingly supports the need to examine the effectiveness of nutritional interventions that may aid in promoting knowledge and supporting LEA understanding and use as a potential tool in prevention.

Athletic nutrition is distinct from general nutritional recommendations, and is often tailored to athlete anthropometrics and energy demands to support athletic performance [25, 26]. For example, dietary recommendations in clinical educational materials delineate athlete meal plans according to training intensity and sport demands [27]. Providing athletes with more detailed and sport-focused information on optimal fueling, macro- and micronutrient ingestion, and ramifications of under-fueling may aid in prevention of LEA. Many studies have implemented educational interventions on nutrition and fueling strategies for athletes to address LEA underlying behaviors. For example, previous research has identified that the inclusion of educational programming describing the implications of nutrition for sport performance and health have successfully increased athlete knowledge of fueling strategies and the negative implications of LEA [28–30]. Previous studies have employed varying modes of educational delivery (remote, in-person), and lengths of interventions, hindering the understanding of optimal approached to such interventions. There is no synthesized information of the effectiveness of past interventions for athletes on nutrition knowledge, attitudes towards fueling, body image, and other key factors underpinning LEA. Compiling the available evidence on interventions and the effects on knowledge and LEA key outcomes is needed to optimize future interventional efforts to disrupt deleterious health and athletic performance effects. Thus, the purpose of this systematic review was to synthesize the available evidence on nutritional interventions to address gaps in nutritional knowledge and combat LEA among athletes.

## Materials and methods

### Search strategy

**Fig 1:**
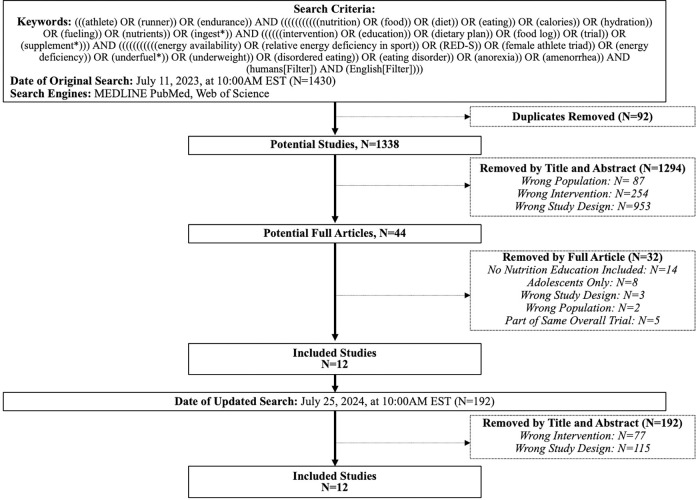
Flowchart depicting the article search criteria and strategy for final article selection.

We conducted a systematic search within Medical Literature Analysis and Retrieval System Online (MEDLINE), PubMed, and Web of Science databases on July 11, 2023, at 10:00 AM EST in accordance with the Preferred Reporting Items for Systematic reviews and Meta-Analyses (PRISMA) guidelines [31]. We performed an updated search on July 26, 2024, at 10:00 AM EST. The search strategy followed Population, Intervention, Comparison, Outcome (PICO) formatting using intentionally broad search terms to identify athletes who underwent nutritional education or dietary interventions and the effect on dietary knowledge, behaviors, and/or LEA/REDs outcomes (Fig 1). Filters were applied to limit studies assessing interventions in humans, and that published in the English language. The search returned 1,430 articles from MEDLINE, PubMed, and Web of Science with 92 duplicate titles. Thus, there were 1,338 total articles to screen. The updated search resulted in 192 additional articles to screen for a total of 1,530 total screened articles.

### Article screening

A single reviewer (AFDL) independently screened articles by title, abstract, and then by full articles. Studies were eligible for inclusion if they a) conducted studies in adult athletes (≥ 18 years of age) to avoid heterogeneity in educational programming for the developing versus skeletally mature athlete, and b) had at least one element of an intervention that included nutritional education. Studies were excluded if they had the wrong study design (e.g., systematic reviews, descriptive studies, case studies), solely reported interventions without nutritional interventions (e.g., dietary supplements or plans, exercise-based programs), reported outcomes solely following intervention without a comparison to baseline timepoints, and/or analyzed the incorrect target population (e.g., only children/adolescents, non-athletes). We opted to include single-arm intervention studies and studies that assessed effects at single time-points (cross-sectional) to identify the full scope of available interventional evidence. Following the three-stage screening, there were 12 articles identified that met the inclusion criteria (Fig 1) [28–30, 32–40].

### Methodological quality

Study methodological quality was assessed by two independent reviewers (AFDL, KEW) using the Physiotherapy Evidence Database (PEDro) scoring criteria [41, 42]. Incongruous scores were rectified by a discussion between the two reviewers and re-review of the manuscript(s) in question. If a consensus could not be reached, a senior author (LMR) would independently review the article to provide the final tie-breaking score, though this was not needed for this review (n = 0).

### Data extraction

Two reviewers extracted study details from all included articles and entered data into a table with contextualizing study details, participant details, interventional details, and key findings. Contextualizing study details were the study design, sample size, and recruitment settings. Participant details were the inclusion/exclusion criteria, and participant sex. Interventional details included the nutrition education delivery mode (i.e., in-person vs. remote), nutrition education components, number, length, and duration of interventions, and additional non-nutrition education methods. Within the nutritional education components, binary assessments were conducted to determine if the interventions: a) were delivered by a registered dietician or other credentialed clinician with training specific to nutrition, b) discussed fueling/caloric intake, c) reviewed macro- and micro-nutrients, d) discussed consequences of under-fueling, e) were tailored to athlete performance needs, and f) if there were interactive components of the intervention. Primary outcomes variables directly related to nutrition knowledge and secondary outcome variables either peripherally related to knowledge interventions (e.g., fueling patterns, patient-reported outcome measures) or related to other study components, and the associated primary and secondary results were obtained from the articles.

### Analyses

Heterogeneity of study samples, nutritional interventions, and outcome measures precluded pooled statistical analyses. Accordingly, only qualitative descriptive assessments were conducted for the included studies.

## Results

The 12 included articles enrolled 719 total participants (mean sample size: 60 athletes; min: 7 athletes [32], max: 107 athletes [33]), and 598 participants completed all study components (mean attrition: 90.2%; min: 63.6% [33], max: 100% [28, 32, 35, 37, 38]). Most interventions were delivered in-person (N = 10; 76.9%) [28, 30, 32, 34–38]. Interventions ranged from single sessions for 10 minutes [28], to 20 sessions each 90–120 minutes [30]. Methodological quality of included studies was overall low as blinding was not feasible for interventions, and there were fairly low retention rates (mean PEDro score: 5; min: 4, max: 6). Full information regarding study context and participant details are offered in Table 1, nutrition educational intervention components in S1 Table, and intervention dosing, additional methods, and outcomes are in S2 Table.

**Table 1:** Article contextualizing study details, and participant details.

Author & Year	Data Extractor Initials and Date of Extraction	Consensus PEDro Score	Study Design	Total Enrolled (N)	Total Analyzed (N)	Attrition (%)	Recruitment Settings	Inclusion/ Exclusion Criteria	Participant Sex
Abood 2000	AFDL; October 2023; Eligible for Inclusion	5	RCT	70	67	95.7%	Division I US University	•Female collegiate divers, cross-country and track runners, swimmers, and softball, basketball, and volleyball athletes	Males: 0% Females: 100%
Brown 2020	AFDL; October 2023; Eligible for Inclusion	5	Quasi-Experimental	24	24	100%	Singular Northwestern US University	• Females 18 years and older enrolled in university dance classes, and dance team members	Males: 0% Females: 100%
Fahrenholtz 2023	AFDL; October 2023; Eligible for Inclusion	4	Non-randomized controlled trial	47	46	97.9%	Norwegian Olympic Sport Centre, Sport Ireland Institute, Swedish National Sport Federation, Swedish sport federations within endurance sports, German Ski Federation, and German Olympic Sports Confederation	• Female tier 3–4 long-distance runners, orienteers, cyclers, cross-country skiers, triathletes, and/or biathlon athletes ages 18–35 years •Currently training ≤5 times/week, • **Exclusion:** Smokers, using hormonal contraceptives, chronic diseases, current pregnancy, menstrual dysfunction unrelated to LEA	Males: 0% Females: 100%
Fredericson 2023	AFDL; October 2023; Eligible for Inclusion	5	Cohort	78	78	100%	Division I US University Cross-Country and Track & Field Programs	•Female middle- and long-distance runners	Males: 0% Females: 100%
Keay 2019	AFDL; October 2023; Eligible for Inclusion	6	RCT	50	45	90%	Cycling Road Race	•Adult male competitive cyclists equivalent to British Cycling category 2+ •Participation in a previous study	Males: 100% Females: 0%
Martin 2020	AFDL; October 2023; Eligible for Inclusion	4	RCT	107	68	63.6%	Collegiate Athletes Across the US	•Currently identify as a collegiate athlete in individual, club, community, or university sports	Males: 46% Females: 54%
Martinelli 2013	AFDL; October 2023; Eligible for Inclusion	5	Cohort	7	7	100%	University Sport Scholars in Italy	•University sports scholars ages 18–23 years •Junior or senior world-class performance level athletes	Males: 43% Females: 57%
Mathisen 2020	AFDL; October 2023; Eligible for Inclusion	6	RCT	149	112	75.2%	Norwegian School of Sport Sciences in Oslo Norway	•Females ages 18–40 years •Diagnosis of bulimia nervosa or binge eating disorder •BMI 17.5–35 kg/m^2^	Males: 0% Females: 100%
Molina-Lopez 2013	AFDL; October 2023; Eligible for Inclusion	5	Cohort	14	14	100%	Puente Genil Handball Club in Granada, Spain	•Handball club athletes	Males: 100% Females: 0%
Perelman 2022	AFDL; October 2023; Eligible for Inclusion	4	RCT	112	79	70.5%	Division II and Division III Universities in Midwestern US	•Male university-sponsored varsity athletes ages ≥18 years •Self-reported body dissatisfaction (>1 on the MBSRQ Overweight Preoccupation, Self-Classified Weight, or Appearance Orientation subscales, or >5 on the Appearance Evaluation subscale or the BAS-2).	Males: 100% Females: 0%
Smith 2008	AFDL; October 2023; Eligible for Inclusion	6	Quasi-RCT	29	29	100%	Division 1 University in Southwestern US	•Female athletes with body dissatisfaction based on pre-study questionnaires •Participation in a previous study	Males: 0% Females: 100%
Yannakoulia 2002	AFDL; October 2023; Eligible for Inclusion	5	Cohort	32	29	90.6%	State School of Dance in Greece	•Dancers ages 18–29 years	Males: 0% Females: 100%

Caption: Study details of included articles for the systematic review, including lead author and year, PEDro scores, study design, participants, attrition, location, inclusion/exclusion criteria, and sex.

Abbreviations: RCT, Randomized Controlled Trial; US, United States; MBRSQ, Multidimensional Body-Self Relations Questionnaire; BAS-2, Body Areas Appreciation Scale; BMI, Body Mass Index (mass in kilograms / height in squared meters).

### Nutritional education intervention components

The interventions were heterogenous across all studies, each with a custom education approach without repeated methodologies between studies. Most included studies had interventions that consisted of education on fueling/caloric intake (83.3%) [28–30, 34–36, 38–40], macro- and micro-nutrients (83.3%) [29, 30, 33–36, 38–40], and were tailored to athlete-specific nutritional needs (91.7%; S1 Table) [28–30, 32–35, 37–40]. Half of included studies (50%) explicitly educated the athletes on ramifications of under-fueling (e.g., REDs, LEA, Female Athlete Triad) [28, 33, 34, 36, 38, 40], and only two studies [38, 40] covered all binary educational intervention features (S1 Table). Several common themes for interactive intervention components were dietary logging [35, 36, 38], and personal reflection on nutrition to support individualized athletic performance [30, 32, 36, 38, 40].

Several studies had concomitant interventions supplementing nutritional education, such as cognitive behavioral education [39] and skeletal loading exercises [29], which may have influenced study findings (S2 Table).

### Outcome measures and main findings

Several studies (n = 5) examined primary outcomes of intervention effects on knowledge tailored to the intervention, and largely reported that knowledge increased following interventions, and compared to control groups who did not receive educational materials (S2 Table) [28, 32, 34, 39, 40]. Additionally, most studies (n = 9) examined primary outcome intervention effects on disordered eating or body image specific questionnaire scores [29, 30, 33–36, 39], with mixed results demonstrating either significant improvements [30, 33, 34, 36, 39] or no significant changes over time (S2 Table) [29, 35]. Several studies (n = 3) examined secondary outcomes of interventional effects on 7-day dietary logs and all identified significantly increased overall caloric intake, and carbohydrate intake (S2 Table) [32, 37, 40]. Additional primary and secondary outcomes are presented in S2 Table.

## Discussion

Timely prevention measures are essential for athletes susceptible to developing LEA to mitigate long-term health outcomes associated with prolonged under-fueling behaviors. Nutritional education is poised to be an impactful preventative measure given that LEA often stems from caloric restriction behaviors, or inadequate understanding of fueling to support athletic involvement [4]. Accordingly, this study aimed to synthesize the existing evidence on nutritional interventions among athletes with the goal of identifying effective educational efforts to combat behaviors underpinning LEA. There were multiple studies that met the systematic review criteria and employed interventions across a range of athletes internationally, with most interventions focused in female populations [28–30, 32–40]. Most studies employed in-person educational sessions that included information on fueling/caloric intake and macro- and micro-nutrients, tailored to athlete-specific nutritional needs. Notably, only half of included studies directly discussed LEA and subsequent health concerns associated with under-fueling. Nutritional intervention approaches were largely heterogeneous, which precluded pooled effect estimates of the effectiveness of programs. Furthermore, there were few studies that reported on consistent outcomes, and thus it is difficult to provide concrete recommendations on best nutritional educations practices. Future research should seek to develop a translational nutritional education plan for broad application in athletes designed to increase nutritional knowledge and combat LEA.

Almost all interventions included education on appropriate fueling behaviors required to support basic physiological functioning and health. Under-fueling is a primary factor contributing to poor energy balance. There is some literature supporting that ingesting a minimum of 45 kcal/kg fat-free mass (FFM) per day is optimal [43], while less than 30 or 25 kcal/kg FFM per day for females and males respectively are the thresholds for LEA [4, 13, 44], though these scores are debated and not substantially validated in male populations [4]. Providing education on fueling requirements and discussing the implications of under-fueling with a nuanced perspective by sex and personal factors to avoid LEA health consequences is an essential component to consider in athlete educational efforts. Fueling-based interventions in tandem with other specific nutritional education elements specific to LEA had a positive influence on self-reported eating attitudes and conceptions as determined with a range of validated (i.e., Eating Disorder Inventory Questionnaire [39], Dutch Eating Behavior Questionnaire [34]) and custom questionnaires when compared to control groups and baseline scores. Thus, the educational efforts served a direct purpose in combating lack of knowledge surrounding optimal energy intake for performance and impacting related disordered eating attitudes.

Perhaps more importantly, multiple studies demonstrated that the educational efforts directly combated behaviors associated with the development of LEA [30, 32–34, 36, 37, 39, 40]. Several studies that employed fueling educational strategies and directly measured fueling ingestion behaviors through dietary logs, most interventions led to athletes significantly increasing their overall total energy intake (small non-responsive cohort in single study [N = 7] [32]) [37, 40]. These interventions were coupled with education tailored to the importance of fueling for supporting athletic participation. Incorporating fueling education serves to directly combat inaccurate messaging and rhetoric that “lean is best,” and instead highlights the importance of adequate energy stores for athletic performance. More evidence is needed to elucidate the benefits of implementing fueling-based interventions on fueling behaviors specific to athletes.

### Micronutrient education

Inadequate micronutrient ingestion as a result of under-fueling has important implications for athlete bone health and injury risk. Vitamin D and Calcium insufficiencies have specifically been linked with poor bone health outcomes among athletes [45, 46]. Indeed, physically active females with LEA often present with reduced blood serum levels of Vitamin D and Calcium, reduced bone mineral density and overall bone mass as determined through dual x-ray absorptiometry, and are at a 4-fold higher risk for bone stress injuries compared to non-LEA counterparts [4, 47, 48]. Several included studies incorporated micronutrient education as part of the overall intervention programming. Unfortunately, only two studies reported on outcomes directly related to micronutrient levels [37] and bone health [29]. It is difficult to examine the direct effects of micronutrient education on the study outcomes given the other educational elements, and, specific to bone health, with parallel exercise-based interventions [29, 37]. Future studies should seek to directly assess the effects of micronutrient education on Vitamin D and Calcium levels for athletes at risk of LEA and micronutrient insufficiency/deficiency.

### Education frequency and delivery mode

The studies examined in this systematic review had wide-ranging session and program durations and were mixed with remote and in-person delivery methods. The study heterogeneity precludes providing a direct recommendation on an ideal education length or method. However, it is promising that both remote and in-person delivery methods led to favorable outcomes specific to each included study. Furthermore, even brief, singular education sessions had promising short-term nutrition knowledge effects [28]. Many clinical or athletic sites where nutritional education efforts would be warranted may not have the capacity to conduct in-person programming. For example, large-scale competitions with high-risk LEA athletes (i.e., competitive cycling, marathons, triathlons) would not necessarily be able to provide in-person sessions for wide-ranging athletes, yet list-serv educational efforts would be more feasible and realistic. Prior in-field research in marathoners suggests that online resources may be a preferred information source for nutrition education among athletes [20]. Based on the included studies, remote education still holds merit for nutrition efforts. Future studies are needed to determine the ideal session length for both short- and long-term nutritional education effects for athletes at risk of developing LEA as prevention efforts to combat LEA development.

### Clinical implications

Although pooled assessments of findings could not be performed due to study heterogeneity, there were emerging themes of successful interventions that may be used to guide clinical interventions in future research and implementation. Studies that demonstrated the most promise for improving athlete nutritional knowledge implemented nutritional education in person for at least two sessions, delivered by credentialed clinicians with content area expertise in sport nutrition. The programs consisted of both nutritional content specific to athletics and introduced LEA and the potential consequences on athletic performance and health. While past research suggests that knowledge alone was not associated with the presence of LEA [15], it is the opinion of the authors that introducing education early may be beneficial in combating LEA and the associated cascade of potential health concerns. Leveraging this evidence-based approach appears to have promise in maximizing resources while achieving the intended effect of increasing knowledge and combating contributors to LEA. Thus, the results of this review support the need for future prospective assessments examining interventions using the suggested evidence-based approach to determine the effects on nutritional knowledge, LEA prevalence, and associated health concerns.

### Limitations

There were several limitations the authors acknowledge for this systematic review. The methodological quality of the included studies was overall low, which was most frequently attributed to lack of blinding, poor retention, and lack of participant randomization. We did not limit studies to assessments of specific nutrition outcomes, which may have influenced the heterogeneity of the included studies. However, there are few published studies that have examined nutritional education interventions, and thus we did not wish to include additional screening parameters related to outcomes in favor of presenting a more complete review of relevant available literature. The included studies were limited to adult athletes and may not be relevant to younger and/or inactive populations. We opted to not report in-depth descriptions of other elements of study interventions (i.e., exercise interventions, micronutrient supplements) of the included articles as this detracted from the primary purpose, yet we acknowledge that additional interventions and study methods were likely to influence the presented studies’ findings. Finally, due to study heterogeneity, pooled analyses were not possible and thus we are unable to provide concrete recommendations on best clinical practices for nutritional education.

## Conclusions

We identified several studies examining the effects of nutritional education efforts related to combating LEA in adult athlete populations. Most studies employed in-person educational sessions that included information on fueling/caloric intake and macro- and micro-nutrients, tailored to athlete-specific audiences. Nutritional intervention approaches and associated outcomes were largely heterogeneous, supporting the need for future research to develop robust nutritional education programming for LEA prevention. 

## Supporting information

S1 Table Study interventional components and nutrition education details. (DOCX)

S2 Table Detailed nutrition education interventional components, primary outcome measures, and main study findings from all included studies. (DOCX)

S3 Table Screened articles and detailed PEDRO scores. Spreadsheet detailing the returned titles for screening, with each tab reflecting the stage of search strategy (article, abstract, full text) and the color-coded reason for exclusion or inclusion (see tab “reason for exclusion key.” The “PEDRO scoring” tab reflects the score for each sub-domain of the scale, and the final scores from each blinded reviewer.
(XLSX)

S1 File PRISMA checklist. Reporting checklist for the systematic review.
(DOCX)
