# Supplementary material for: Nutrition educational interventions for athletes related to low energy availability: A systematic review
Source: PLoS One. 2025 Feb 14;20(2):e0314506. doi: 10.1371/journal.pone.0314506 (PMC11828352; doi:10.1371/journal.pone.0314506)
Supplement: S1 Table — (DOCX) [file pone.0314506.s001.docx]

**Supplementary Table 1.** Nutritional education intervention components.

| **Author & Year** | **Delivered by Nutritionist/ Dietician?** | **Fueling/ Caloric Intake?** | **Macro-/ Micro-nutrients?** | **Consequences of Under-fueling?** | **Athlete- Specific Nutrition?** | **Inter-active?** | **Nutrition Education Intervention Details** |
| --- | --- | --- | --- | --- | --- | --- | --- |
| Abood 2000 |  | **X** | **X** |  | **X** |  | **Nutrition knowledge and nutrition beliefs/myths related to athletic performance:**  - Caloric needs of the female athlete, distribution of energy nutrients, use of supplements  - Nutrition beliefs and myths: guidelines for healthy approaches to weight management |
| Brown 2020 | **X** | **X** |  | **X** | **X** |  | **Nutrition:**  Registered Dietician discussion on fueling strategies to promote optimal dietary intake and athletic performance.  **Female Athlete Triad:**  - Etiology and symptoms  - Testimonies from former athletes  -Discussion led by a college coach on pressures athletes may feel to look a certain way or to achieve a certain body weight and composition. |
| Fahrenholtz 2023 | **X** | **X** | **X** | **X** | **X** | **X** | **Sports Nutrition:**  - Macronutrients  - Meal patterns, fueling strategies  - Micronutrients and supplements  - Body weight & composition  - Nutrition for recovery when injured  - Consultations using motivational interviewing based on self-determination theory and transtheoretical theory of health behavior change (goal setting, problem solving, review behavior goal, commitment, feedback on behavior, social support)  **REDs:**  - Causes, health and performance consequences  - Menstrual cycle & performance |
| Fredericson 2023 | **X** | **X** | **X** | **X** | **X** | **X** | **Pre-season Team Education:**  -Emphasise importance of adequate caloric intake and caloric timing for health and performance  **Annual Individual Assessment and Education:**  - Evaluate diets  -Develop 1-2 nutrition goals based on current training to optimise energy intake and bone-building nutrients  -Optional follow-up sessions based Female Athlete Triad risk; optional phone app with additional running-specific nutrition information |
| Keay 2019 | **X** | **X** | **X** |  | **X** |  | **Nutritional advice aimed to maintain adequate energy availability**  - Recommendations for general nutrition and fueling around training sessions  -Recommended 1000 IUs vitamin D supplementation |
| Martin 2020 |  |  |  | **X** | **X** | **X** | **Athletic Nutrition:**  -Open discussion formats on "imbalances in eating, exercise, hydration, and mental health" that can occur among collegiate athletes and how to seek help for them.  - Role-play activity, and sigma reduction video  **Eating Pathologies:**  -Psychoeducation on eating pathologies and treatment among collegiate athletes  -Stigma reduction of eating pathologies and treatment in collegiate athletes  - Interactive workshop with didactic psychoeducation |
| Martinelli 2013 | **X** | **X** | **X** |  | **X** | **X** | **Sports Nutrition:**  -Introduction to sports nutrition  -Fueling  -Interpreting diet analysis  -Optimizing hydration  -Recovery nutrition  -Protein & supplements  -Tailored assessment/application for athlete needs |
| Mathisen 2020 | **X** | **X** | **X** |  | **X** | **X** | **Dietary routines & Structure**  -Meal frequency  -Portions  -Eating situation  -Exercise theory  **Nutritional knowledge and practical skills**  -Energy needs based on daily routines  -Nutrients  -Nutritional labels  -Impulsive food shopping  -Exercise theory  -Sports nutrition  **Reflections and Future Dietary Planning** |
| Molina-Lopez 2013 | **X** |  | **X** |  | **X** |  | -Types of nutrients and their importance for maintaining good health in basically healthy persons  -Specific nutritional requirements in physically active individuals  -Frequent nutritional errors in physically active individuals |
| Perelman 2022 |  | **X** | **X** | **X** |  | **X** | **Educational Components**  -Consequences of RED-S, defining body ideals  -Healthy and unhealthy exercise behaviors  -Gradual lifestyle changes to balance energy intake/output  -Prioritization of nutrient-dense foods  **Interactive Components**  -Food logging  -Goal setting  -Homework focused on positive self-talk |
| Smith 2008 |  | **X** | **X** |  | **X** | **X** | - Attitudes towards eating, body image, and sport performance  -Energy balance  -Nutritional basics  -Food and exercise balance  -Food logging |
| Yannakoulia 2002 |  | **X** | **X** | **X** | **X** |  | **General Nutrition Education**  -Role of nutrition  -Energy balance  -Body composition  -Nutrients  -Balanced diet  -Meal patterns  **Primary Prevention of Eating Disorders**  -Body shape, standards, image  **Nutrition for Dancers**  -Eating & emotions  -Food misconceptions  -Fluids  -Weight loss  -Aerobic exercise  -Meal patterns |

*Caption: Study interventional components and nutrition education details.*
