# Supplementary material for: Nutrition educational interventions for athletes related to low energy availability: A systematic review
Source: PLoS One. 2025 Feb 14;20(2):e0314506. doi: 10.1371/journal.pone.0314506 (PMC11828352; doi:10.1371/journal.pone.0314506)
Supplement: S2 Table — (DOCX) [file pone.0314506.s002.docx]

**Supplementary Table 2.** Intervention dosing, additional methods, and outcomes.

| **Author & Year** | **Intervention Delivery Method** | **Additional Methods** | **# and Length of Sessions** | **Session Frequency and Duration** | **Primary Outcome Measures** | **Primary Findings** | **Secondary Outcome Measures** | **Secondary Findings** |
| --- | --- | --- | --- | --- | --- | --- | --- | --- |
| Abood 2000 | In-person | **Additional Educational Components:**  -Self-esteem  -Performance pressure  -Stress management  **Control Group:**  - Study hall | 8; 60 mins | Weekly; 2 Months | - Drive for Thinness and Body Dissatisfaction EDI-2 subscales  - Nutrition knowledge | - Significantly decreased Drive for Thinness and Body Dissatisfaction EDI-2 Subscales compared to baseline and control  - Control group experienced a decrease in nutrition knowledge | -SAS  -SCAT  -RSES | - Control group experienced a decrease in self-esteem  -No other significant differences |
| Brown 2020 | In-person | n/a | 1; 10 mins | 1; 1 | - Female Athlete Triad knowledge questionnaire | - Knowledge increased 32% from pre- to post-intervention | - | - |
| Fahrenholtz 2023 | Remote | **Control Group:**  -No intervention; waiting list to optionally receive intervention after study | 24; 15-43 mins | ~Weekly; 4 Years | -Sports nutrition knowledge questionnaire  -Sports nutrition-related behavior questionnaire  -Dietary intake via 7-day food log | -Significantly improved sports nutrition knowledge compared to baseline and the control group  -Significantly increased carbohydrate and total energy intake compared to the control group and baseline | - | - |
| Fredericson 2023 | In-person | n/a | ~8; 15-30 mins | 2; 6 Months | -Bone stress injuries | -No risk reduction for BSIs in the intervention phase compared to the observation phase overall  -Fewer trabecular-rich bone BSIs during the intervention phase  -1 institution had reduced BSI risk compared to other institution during intervention phase | - | - |
| Keay 2019 | Remote | **Additional Intervention Group:**  Skeletal loading exercises | DNS; DNS | Weekly to Monthly; 6 Months | -SEAQ-I | -Most individuals had no change in their SEAQ-I scores, with only 12 reporting positive changes | -Sport-specific performance measures  -Bone health measures  -Endocrine health measures | **Those who followed both interventions (cross-over evident):**  - Increased 2% lumbar spine BMD  -Increased vitamin D concentration  - Improved cycling performance |
| Martin 2020 | Remote | **Control condition:** Webinar on sports hydration | 1; 17 mins | 1; 1 | - ATSPPH-S  -GHSQ  -SSOSH  -Help-seeking behavior survey | -Significantly increased attitudes towards seeking help for eating pathologies and general mental health concerns post-treatment compared to baseline and controls.  -No significant decreases in perceived social stigma towards seeking help for mental health problem. | - EDE-Q at baseline  -Subjective Intervention acceptability (1-10) | - 22.4% of sample at risk for an eating pathology (>90th percentile of EDE-Q) at baseline -Average workshop rating was favourable at 8.89 |
| Martinelli 2013 | In-person | n/a | 6; DNS | Weekly; 5 Months | -Nutrition knowledge questionnaire  -7-day food diary | -No significant changes in dietary intake pre- to post-intervention  -Nutrition knowledge significantly increased pre- to post-intervention | - | - |
| Mathisen 2020 | In-person | **Additional Intervention:** Exercise therapy  **Comparison group:** Cognitive behavioral therapy | 20; 90-120 mins | ~Weekly; 4 Months | -EDE-Q 6.0  -CIA 3.0  -BDI-Ia  -SWLS | -Significantly improved EDE-Q global score and body weight concern sub-scale at post-intervention compared to baseline and the CBT control group.  -Significantly improved CIA 3.0 and SWLS scores compared to baseline but not compared to CBT group. | - | - |
| Molina-Lopez 2013 | In-person | n/a | DNS; DNS | 3 Phases; 2 Months | -Estimated energy intake  -Macronutrient intake  -Consumption frequency | - Mean estimated energy intake was significantly greater at follow-up  -Carbohydrate intake was significantly higher at follow-up  -Fat intake was significantly higher at follow-up | -Blood panels | -No significant differences at follow-up for glucose, creatinine, iron, transferrin, prealbumin, HDL, LDLs, triglycerides, total cholesterol |
| Perelman 2022 | In-person | n/a | 3; 80 mins | Weekly; 3 Weeks | - MBSRQ  -DMS  -SATAQ-4  -MDI | -All primary outcome measures were significantly improved after the 3-week intervention, and were maintained at 1-month follow-up | - | - |
| Smith 2008 | In-person | **Other Intervention Arm:**  - Cognitive-dissonance intervention  -Demystifying thin is ideal body image vs. healthy body for performance  -Self-affirmations  -Identifying pressures/barriers and strategies to overcome stigmas  **Control Arm:**  No interventions | 3; 60 mins | Weekly; 3 Weeks | -Beliefs About Attractiveness Scale  -BSS  -BSQ  -PANAS  -BULIT-R  -Dutch Restrained Eating Scale | No significant differences for the nutrition education intervention group over time or compared to the other study groups | Within-group findings for the other intervention arm, and control arm | **Other Intervention Arm:**  Significantly less internalization, and increased body dissatisfaction over time  **Control Arm:**  Significantly more internalization, and increased body dissatisfaction over time. |
| Yannakoulia 2002 | In-person | n/a | 12; 120 mins | Weekly; 3 Months | -EAT-26  -DEBQ  -Nutrition Knowledge Test  -BSS | -Significant decrease in EAT-26 scores and dieting sub-scale at follow-up  -Significant decrease in DEBQ restraint subscale at follow-up | - | - |

*Caption: Detailed nutrition education interventional components, primary outcome measures, and main study findings from all included studies.*

*Abbreviations: EDI-2), Eating Disorder Inventory-2; SAS, Self-Rating Anxiety Scale; SCAT, Sport Competition Anxiety Test; RSES, Rosenberg Self-Esteem Scale; BSI, Bone Stress Injury; DNS, Did Not Specify; ATSPPH-S, Attitudes Toward Seeking Professional Psychological Help-Short Form; SEAQ-I, Sport-specific Energy Availability Questionnaire; GHSQ, General Help Seeking Questionnaire; SSOSH, Self-stigma of Seeking Help Scale; EDE-Q 6.0, Eating Disorder Examination Questionnaire Version 6; CIA 3.0, Clinical Impairment Assessment Version 3; BDI-Ia, Beck Depression Inventory; SWLS, Satisfaction With Life Scale; HDL, High-Density Lipoproteins; LDL, Low Density Lipoproteins; MBSRQ, Multidimensional Body-Self Relations Questionnaire-Appearance Scales; DMS, Drive for Muscularity Scale; SATAQ-4, Sociocultural Attitudes Towards Appearance Questionnaire-4-Revised; MDI, 27-item Muscle Dysmorphia Inventory; BSS, Body Parts Satisfaction Scale; BSQ, Body Shape Questionnaire-revised; PANAS, Positive Affect and Negative Affect Scale-Revised; BULIT-R, Bulimia Test-Revised; EAT-26, Eating Attitudes Test; DEBQ, Dutch Eating Behavior Questionnaire.*
